# Obinutuzumab for Rituximab-Intolerant Minimal Change Disease: A Case Report and Review of Recent Literature

**DOI:** 10.7759/cureus.114295

**Published:** 2026-08-10

**Authors:** Hui Yi Shan

**Affiliations:** 1 Medicine/Nephrology and Hypertension, Keck School of Medicine of University of Southern California, Los Angeles, USA

**Keywords:** adult minimal change disease, frequently relapsing minimal change disease, minimal change disease, obinutuzumab, rituximab, steroid-dependent minimal change disease

## Abstract

B lymphocytes play a central role in the pathogenesis of minimal change disease (MCD). Rituximab is a chimeric (murine-human) type I anti-CD20 monoclonal antibody that depletes B cells and is used in the management of frequently relapsing (FR) and steroid-dependent (SD) MCD. However, continued treatment with rituximab is not feasible in some patients because of severe infusion reactions or hypersensitivity.

Obinutuzumab is a humanized type II anti-CD20 monoclonal antibody that produces potent B-cell depletion and has been used as an alternative B-cell-depleting therapy in selected immune-mediated diseases. Experience with its use in adults with FR/SD MCD remains limited.

The author reports an adult patient with MCD who developed an immediate hypersensitivity reaction to rituximab, confirmed by positive skin testing, which precluded further treatment. The patient subsequently received obinutuzumab without adverse reactions and achieved clinical remission. To the author's knowledge, this represents one of the first reported cases of successful obinutuzumab treatment following confirmed rituximab allergy in an adult with MCD. Although based on a single case, this report suggests that obinutuzumab may represent a potential therapeutic alternative for selected patients with MCD who are unable to receive rituximab because of hypersensitivity. The current literature on the use of obinutuzumab in adult MCD is also reviewed.

## Introduction

Minimal change disease (MCD) accounts for approximately 10-15% of nephrotic syndrome in adults [1]. Although most patients initially respond to corticosteroids, many develop steroid-dependent or frequently relapsing disease requiring prolonged immunosuppressive therapy. Because repeated corticosteroid exposure is associated with substantial toxicity, effective steroid-sparing therapies are essential.

Rituximab has become an established treatment for steroid-dependent (SD) and frequently relapsing (FR) MCD, producing high remission rates and prolonged relapse-free survival. However, infusion reactions, immediate hypersensitivity, and anti-drug antibodies may limit repeated administration in a subset of patients. Effective therapeutic alternatives for patients who cannot continue rituximab are not well defined.

Obinutuzumab is a glycoengineered humanized type II anti-CD20 monoclonal antibody that induces more potent and sustained B-cell depletion than rituximab. Although it has shown promising results in several immune-mediated kidney diseases such as lupus nephritis and membranous nephropathy, experience in MCD is limited [2,3]. The author reports a patient with steroid-dependent MCD who developed confirmed rituximab hypersensitivity and was successfully treated with obinutuzumab, resulting in remission without recurrent infusion-related reactions.

## Case presentation

A 36-year-old woman presented in April 2024 with bilateral leg swelling, an 18-pound weight gain over 10 days, and nephrotic-range proteinuria, as evidenced by a urine protein-to-creatinine ratio (UPCR) of 4.5 g/g (reference range, <0.15 g/g). Serum creatinine was 0.93 mg/dL (reference range, 0.51-0.95 mg/dL), and serum albumin was 3.3 g/dL (reference range, 3.5-5.2 g/dL). A kidney biopsy established the diagnosis of minimal change disease. Treatment with prednisone at a dose of 60 mg daily was initiated and continued for two months, resulting in complete remission. The dose was then gradually tapered.

In September 2024, while the prednisone dose was reduced to 20 mg daily, UPCR increased to 5.4 g/g. Prednisone was increased to 60 mg daily. After one month of treatment, UPCR remained elevated at 5.8 g/g.

In November 2024, rituximab was administered intravenously at a dose of 1 g, followed by a second 1 g infusion two weeks later. During the initial infusion, the patient developed a choking sensation, which resolved after treatment with glucocorticoids and antihistamine. Proteinuria subsequently declined, and complete remission was achieved in December 2024.

A second relapse occurred in November 2025, with UPCR increasing to 2.7 g/g. A single 1 g dose of rituximab was administered on November 5, 2025. The patient again felt a choking sensation, coughing, and dysphagia during the infusion, which subsided after 30 minutes. After the infusion, the patient developed severe persistent headache, nausea, and vomiting. Allergy evaluation, including rituximab skin testing, was positive, confirming a hypersensitivity reaction. Although proteinuria decreased to approximately 0.5 g/g after treatment, it remained stable at that level for four months without further decline.

**Figure 1:**
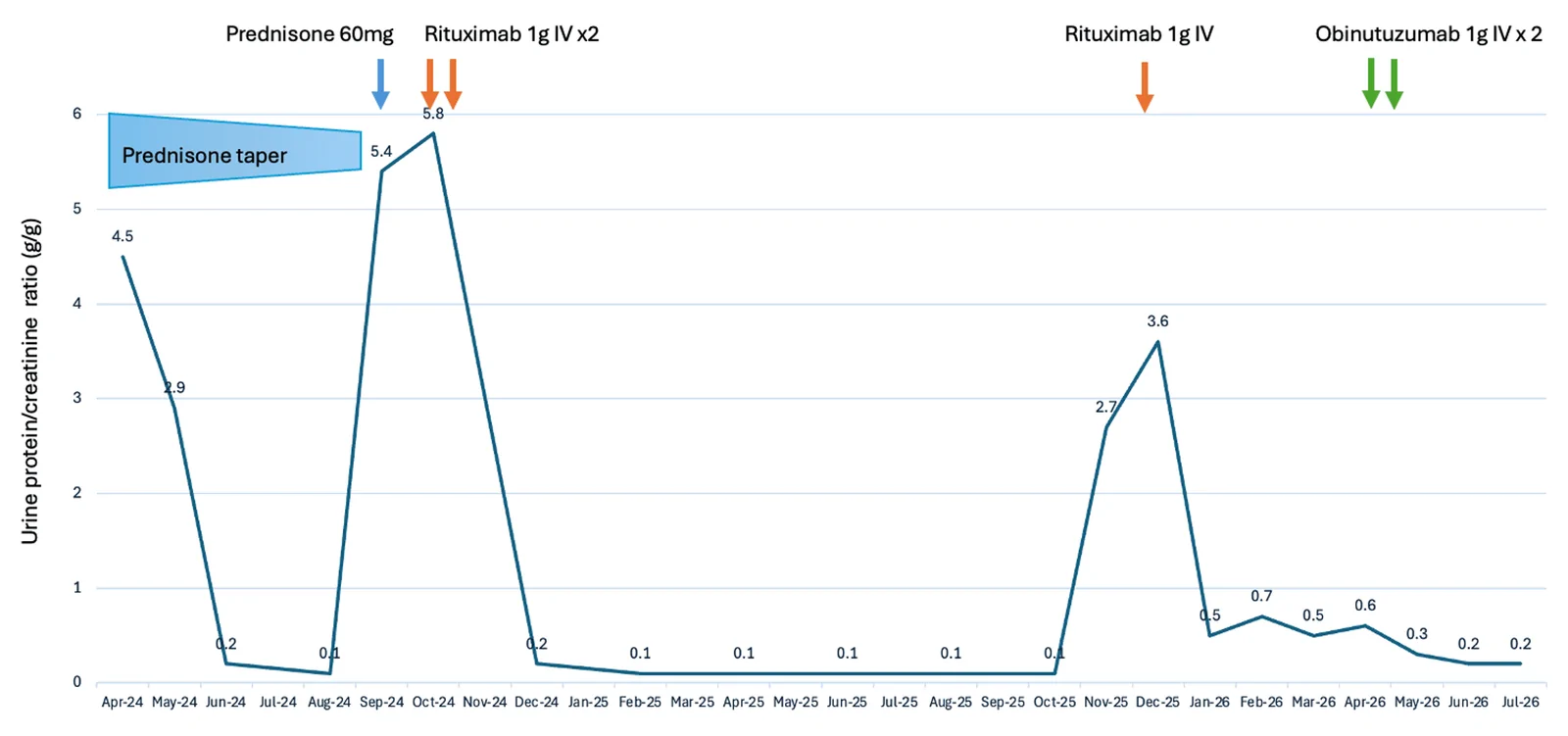
Patient’s Treatment History and Proteinuria Trend

Because of the confirmed rituximab allergy and persistent residual proteinuria, treatment was changed to obinutuzumab. The patient received two intravenous doses of obinutuzumab, 1 g each administered on April 9 and April 23, 2026, with premedication consisting of methylprednisolone 100 mg IV, acetaminophen 1,000 mg orally, and diphenhydramine 50 mg IV given 30 minutes before each infusion. Treatment was well tolerated without infusion-related adverse events or infectious complications. The patient was followed monthly with urine protein-to-creatinine ratio (UPCR) monitoring. Two months after obinutuzumab treatment, UPCR decreased from 0.6 g/g to 0.2 g/g, and the proteinuria remained at 0.2 g/g three months post-therapy (Figure 1). Serum creatinine and albumin levels were normal at 0.71 mg/dL and 3.8 g/dL, respectively, in July, 2026.

## Discussion

Rituximab has become an effective steroid-sparing therapy for patients with steroid-dependent or frequently relapsing minimal change disease (MCD). A recent meta-analysis of observational studies reported a pooled remission rate of 89% (95% CI, 83%-94%), although significant between-study heterogeneity was observed (I² = 62%), likely reflecting differences in study design, patient populations, rituximab dosing regimens, and duration of follow-up [4]. In addition, a randomized controlled trial demonstrated a relapse-free rate of 87.4% at 49 weeks compared with 38.0% in the placebo group [5]. Maintenance rituximab therapy has also been shown to further prolong relapse-free survival [6].

Despite its efficacy, some patients develop infusion-related hypersensitivity reactions or anti-rituximab antibodies (ARAs), limiting repeated treatment. ARAs may reduce efficacy by accelerating B-cell reconstitution and increasing relapse risk. In a randomized trial by Isaka et al., ARAs developed in 15.6% of rituximab-treated adults with frequently relapsing or steroid-dependent nephrotic syndrome, highlighting the need for alternative anti-CD20 therapies [5].

Obinutuzumab is a glycoengineered humanized type II anti-CD20 monoclonal antibody that produces greater antibody-dependent cellular cytotoxicity, enhanced direct B-cell death, and more sustained B-cell depletion than rituximab. In patients with lupus nephritis, Looney et al. demonstrated higher rates of sustained B-cell depletion with obinutuzumab than rituximab (93% vs. 63% at 24 weeks) [7]. Although MCD has a distinct pathogenesis and direct comparative data between obinutuzumab and rituximab in MCD are lacking, relapse frequently coincides with B-cell recovery following anti-CD20 therapy, suggesting that more durable B-cell depletion may help prolong remission. In addition, humanized antibodies are generally associated with lower immunogenicity than chimeric antibodies, and obinutuzumab demonstrates minimal cross-reactivity with anti-rituximab antibodies, supporting its potential use in patients with rituximab hypersensitivity or anti-rituximab antibodies [8,9].

In this case, obinutuzumab successfully maintained remission in a patient with steroid-dependent MCD who had previously responded to rituximab but developed a hypersensitivity reaction that precluded further treatment. The patient tolerated obinutuzumab without infusion-related adverse events.

To the best of the authors’ knowledge, this represents one of the first reported cases of successful obinutuzumab use in an adult patient with MCD following confirmed rituximab hypersensitivity. A literature search of PubMed/MEDLINE and Embase identified limited reports of obinutuzumab use in adult MCD, primarily involving patients with rituximab resistance or inadequate response, but no previously reported cases describing a transition to obinutuzumab specifically because of confirmed rituximab allergy. Beyond this case, emerging evidence supports the efficacy of obinutuzumab in patients with MCD who have failed rituximab therapy. Lin et al. reported remission in all eight patients with MCD treated with obinutuzumab after rituximab failure, while a recent retrospective study demonstrated longer relapse-free survival with obinutuzumab compared with rituximab, with 89.7% of rituximab-resistant patients achieving remission following obinutuzumab therapy [10,11].

## Conclusions

This case adds to the emerging evidence supporting obinutuzumab as an alternative anti-CD20 therapy for adult patients with steroid-dependent or frequently relapsing minimal change disease (MCD) who are unable to continue rituximab because of hypersensitivity. In this patient, obinutuzumab was well tolerated despite confirmed rituximab allergy and was followed by further reduction in proteinuria and clinical remission. Its greater and more sustained B-cell depletion and minimal cross-reactivity with anti-rituximab antibodies provide a biologic rationale for further evaluation in patients with MCD.

The findings of this report should be interpreted within the limitations of a single-patient case design and limited duration of follow-up. Although the patient's clinical course was encouraging, this report alone cannot establish the efficacy of obinutuzumab for the treatment of MCD. Further studies are needed to define the role of obinutuzumab in MCD, including prospective evaluation of optimal dosing strategies, comparative efficacy with rituximab, durability of remission, and long-term safety monitoring in the setting of repeated B-cell depletion.
